# Evidence of longitudinal differences in spring migration strategies of an Arctic‐nesting goose

**DOI:** 10.1002/ece3.11665

**Published:** 2024-09-02

**Authors:** Jay A. VonBank, Kevin J. Kraai, Daniel P. Collins, Paul T. Link, Mitch D. Weegman, Lei Cao, Bart M. Ballard

**Affiliations:** ^1^ U.S. Geological Survey, Northern Prairie Wildlife Research Center Jamestown North Dakota USA; ^2^ Caesar Kleberg Wildlife Research Institute Texas A&M University – Kingsville Kingsville Texas USA; ^3^ Texas Parks and Wildlife Department Canyon Texas USA; ^4^ U.S. Fish and Wildlife Service Albuquerque New Mexico USA; ^5^ Louisiana Department of Wildlife and Fisheries Baton Rouge Louisiana USA; ^6^ Department of Biology University of Saskatchewan Saskatoon Saskatchewan Canada; ^7^ State Key Laboratory of Urban and Regional Ecology, Research Center for Eco‐Environmental Sciences Chinese Academy of Sciences Beijing China; ^8^ University of Chinese Academy of Sciences Beijing China

**Keywords:** biotelemetry, Greater White‐fronted Goose, hidden Markov model, migration, nesting propensity

## Abstract

During spring, migratory birds are required to optimally balance energetic costs of migration across heterogeneous landscapes and weather conditions to survive and reproduce successfully. Therefore, an individual's migratory performance may influence reproductive outcomes. Given large‐scale changes in land use, climate, and potential carry‐over effects, understanding how individuals migrate in relation to breeding outcomes is critical to predicting how future scenarios may affect populations. We used GPS tracking devices on 56 Greater White‐fronted Geese (*Anser albifrons*) during four spring migrations to examine whether migration characteristics influenced breeding propensity and breeding outcome. We found a strong longitudinal difference in arrival to the breeding areas (18 days earlier), pre‐nesting duration (90.9% longer), and incubation initiation dates (9 days earlier) between western‐ and eastern‐Arctic breeding regions, with contrasting effects on breeding outcomes, but no migration characteristic strongly influenced breeding outcome. We found that breeding region influenced whether an individual likely pursued a capital or income breeding strategy. Where individuals fell along the capital‐income breeding continuum was influenced by longitude, revealing geographic effects of life‐history strategy among conspecifics. Factors that govern breeding outcomes likely occur primarily upon arrival to breeding areas or are related to individual quality and previous breeding outcome, and may not be directly tied to migratory decision‐making across broad scales.

## Graphical abstract

During spring, migratory birds are required to optimally balance energetic costs of migration across heterogeneous landscapes and weather conditions in order to survive and reproduce successfully. Therefore, an individual's migratory performance may influence reproductive outcomes. We used GPS tracking devices on Greater White‐fronted Geese during spring migration to examine whether migration characteristics influenced breeding propensity and breeding outcome. We found highly variable migration strategies led to successful reproductive outcomes, and a geographic gradient of migration characteristics influenced where individuals fell along the capital‐income breeding continuum, revealing geographic effects of life‐history strategy among conspecifics.

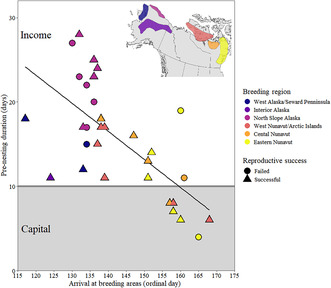

## 1 INTRODUCTION

During spring migration, individuals balance the energetic cost of migration with the nutrient and energy requirements of molt, thermoregulation, and social activities (e.g., courtship and maintaining pair bonds) which must be optimally managed to survive, complete spring migration, and successfully breed (Arzel et al., 2006; Klaassen, 2003; Stafford et al., 2014). Birds that arrive early to spring stopover locations and breeding areas potentially benefit if they can exploit food resources and secure favorable nesting locations, but early migrants have a higher probability of facing adverse and irregular spring weather conditions potentially affecting their fitness (Alerstam, 2011). Changing climatic conditions can influence resource quality and timing of resource availability at stopover locations and breeding areas, particularly for species breeding in the Arctic where storing nutrient reserves is often imperative for successful breeding (Drent et al., 2007; Spragens et al., 2015; Prop et al., 2003; van der Graaf, 2006). Climate change has primarily affected Arctic‐nesting geese through phenological mismatch during the breeding season, but also through warming sea temperatures, increasing and variable precipitation, and miscued migratory movements (Aubry et al., 2012; Boyd & Fox, 2008; Clausen & Clausen, 2013). Recent evidence suggests that goose species are capable of shortening migration duration to match spring phenology; however, increasing migration speed decreases the time available to be spent at stopovers refueling energy, which would otherwise be reserved for egg laying and incubation (Lameris et al., 2018). Geese are then required to increase time spent replenishing reserves in breeding areas instead of during migration, which further delays nesting and still induces a phenological mismatch (Lameris et al., 2018). Given the differences in biotic and abiotic conditions experienced by individuals in wintering areas (e.g., disturbances, body condition, and weather), during migration (e.g., availability and quality of stopover sites along migratory routes and distances traveled), and on breeding areas (e.g., differential effects of climate change across the breeding range), there are undoubtedly varying strategies that can result in successful breeding. Quantifying spring migration characteristics may allow insight into how migration strategy affects reproductive success.

In North America, Greater White‐fronted Geese (*Anser albifrons frontalis*, hereafter White‐fronted Geese) form two distinct populations with little interchange. The Pacific population breeds on the Yukon‐Kuskokwim Delta and Bristol Bay lowlands in Alaska and winters in California and western Mexico. The Midcontinent population breeds from the Bering Sea to the central Canadian Arctic and west coast of Hudson Bay, and migrates through the Central and Mississippi Flyways to winter from Kansas to Louisiana, Texas, and Mexico. Over the last 20 years, Midcontinent White‐fronted Geese have shifted their core wintering range more than 300 km northeastward to the Mississippi Alluvial Valley (MAV) in Arkansas and Mississippi, with unknown consequences to spring migration strategies (Moore et al., 2023). Body condition of White‐fronted Geese leaving wintering areas is known to vary among individuals (Ely & Raveling, 1989; Massey et al., 2019), and food available to accumulate endogenous stores throughout spring migration is directly affected by migration timing (Budeau et al., 1991). For instance, Massey et al. (2019) found that White‐fronted Geese in the MAV begin spring migration with smaller lipid stores than when geese arrived in autumn. Similarly, VonBank et al. (2021) found that White‐fronted Geese wintering in the MAV expended more energy per day than individuals wintering in most other areas, including the Texas Gulf Coast, Rolling/High Plains of Texas, and Chenier Plain of Texas and Louisiana, within the Midcontinent region. Wintering in areas with greater energy expenditure may force individuals to begin migration with smaller body reserves, and may affect subsequent reproductive success if geese cannot counterbalance that deficit during migration, demonstrating a carry‐over effect from winter (Sedinger & Alisauskas, 2014).

Migration characteristics, such as migration chronology, migration distance, and number and location of stopovers, have not been investigated or described holistically at the population level of Midcontinent White‐fronted Geese due to the extensive spatial scale of wintering, migration, and breeding ranges. Midcontinent White‐fronted Goose winter distributions have shifted northward, and characteristics of spring migration have likely concomitantly changed, with unknown implications on reproductive outcomes. Previous research indicates disparities in migration timing and stopovers during spring migration in White‐fronted Geese based on breeding geography. Taiga‐breeding White‐fronted Geese are thought to initiate spring migrations earlier and are more likely to stopover in certain geographic areas, such as the Rainwater Basin of Nebraska, than tundra‐breeding geese (Ely et al., 2013; Webb, 2006). Recent changes to winter distribution and timing of breeding have unknown effects on how White‐fronted Geese use traditional stopover areas. Thus, an understanding of migration timing and routes will help conservation planners identify key stopover sites during spring migration.

We examined whether spring migration characteristics influenced breeding outcomes across the entire breeding range of Midcontinent White‐fronted Geese. The objectives of this study were to (1) describe spring migration characteristics in Midcontinent White‐fronted Geese, (2) determine the spatial extent and key stopover areas during spring migration, (3) determine if migration characteristics influence breeding outcomes (i.e., attempt/deferral and success/failure) at the population level and between geographic breeding regions, and (4) investigate where individuals fell along the capital‐income breeding strategy continuum (Klaassen et al., 2006). We hypothesized that shorter migration distances and earlier arrival to breeding areas would increase breeding propensity. Additionally, we tested migration duration to determine if White‐fronted Geese exhibited a time‐minimizing (i.e., shorter migration duration) or energy‐minimizing (i.e., more days, more stopovers, and shorter migratory movements) migration strategy. Given the recent winter distribution shift, we hypothesized that geese beginning migration in the MAV, the current primary wintering region, would have a higher rate of breeding attempts compared to geese wintering in historical regions if carry‐over effects from winter exist. Despite geese expending more energy in the MAV during winter, they also feed at higher rates than other winter regions (VonBank et al., 2021) which may increase pre‐migration body condition. The MAV is generally farther north than other wintering regions which may benefit geese by shortening migration distance and/or duration. Furthermore, we hypothesized that geese that spent more time at stopovers of high‐use at the population level would have an increased probability of attempting to breed, assuming that these areas contain higher abundance or quality of food resources (Fretwell & Lucas, 1969). Additionally, we hypothesized that geese that initiated nests earlier would be more successful than later nesting geese due to time‐constraints associated with breeding at high latitudes such as the Arctic (Lepage et al., 2000).

## 2 METHODS

### 2.1 Goose capture, devices, and tracking

From October to February in 2016–2019, we captured White‐fronted Geese using rocket nets and deployed 171 global positioning system (GPS) tracking devices in Texas (*n* = 89) and Louisiana (*n* = 82) across varying ecoregions important to wintering White‐fronted Geese (VonBank, 2020). We deployed three models of Cellular Tracking Technologies devices (BT3.0, BT3.5, BT3.75, 7.2 m accuracy, Rio Grande, New Jersey, USA) and two models of Ornitela devices (OrniTrack N‐38 and N‐44, 6.5 m accuracy, Vilnius, Lithuania) which all used Global System for Mobile communication (GSM) cellular data transmission, were solar‐rechargeable, and were attached as a neckband. Tracking devices were set to record GPS locations every 15 or 30 min, but occasionally individual devices were changed to less frequent locations. However, those individuals were removed to retain devices with consistent location collection schedules (see Section 2.2). We determined sex and age of individuals using cloacal inversion and plumage features (e.g., black‐barring on breast and white feather patch behind bill), and fit devices only to after‐hatch‐year females or males per capture (specifically targeting females) to eliminate potential bias of non‐independent data from a mated pair due to long‐term pair bonds in geese (Black, 1996).

### 2.2 Data preparation

We subset all GPS locations from 15 January to 1 August for each year and individual to include the pre‐migration, migration, nesting, brood‐rearing, and wing molt periods to ensure the entire migratory and breeding periods were included. We visually assessed all movement trajectories for and removed erroneous locations due to GPS error, removed all locations with horizontal dilution of precision >5 (VonBank et al., 2020, 2021), and only included individuals that contained sufficient GPS data to determine breeding status and thus successfully completed migration; only complete migrations were included. Additionally, we removed individuals who experienced complete or partial transmitter failure, experienced mortality during migration (e.g., predated), or were harvested during the hunting season prior to spring migration. We resampled devices set for 15‐min duty cycles to 30‐min intervals for consistency among individuals and removed individuals with varying GPS schedules. We calculated net squared displacement (Bunnefeld et al., 2010) to determine the first migratory movement northward away from wintering areas (i.e., migration start) that was ≥50‐km displacement (Drent et al., 2007), and retained GPS data beginning 7 days prior to migration start through 1 August each year.

### 2.3 Movement models

We used dynamic Brownian bridge movement modeling (dBBMM) to identify spring migration routes and quantify space use by White‐fronted Geese at the population level (Kranstauber et al., 2012). The dBBMM creates a spatial probability density function (i.e., a utilization distribution, UD) between successive locations, which includes Brownian motion variance ($\sigma_{m}^{2}$) for each segment along the movement track (Kranstauber et al., 2012). The dBBMM can readily handle missing locations and allow inference about animal behavior by reducing subjective user input previously used to identify behavioral segments (e.g., migratory vs. non‐migratory). These features allow the identification of high‐use areas (i.e., stopover locations) to be distinguished from low‐use areas (i.e., migratory flight paths), identified through the UD.

**FIGURE 1:**
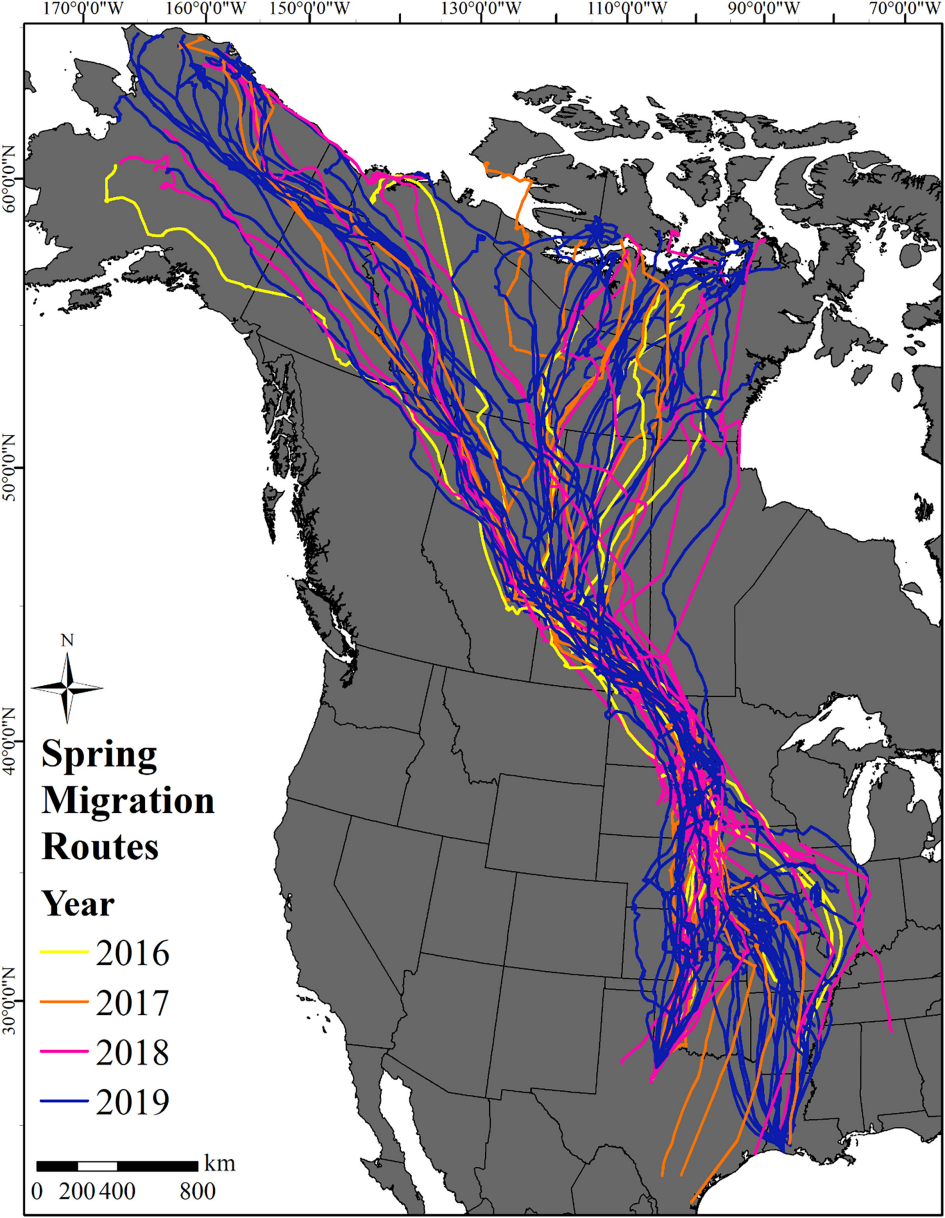
Spring migration tracks from 57 global positioning system (GPS) tagged Greater White‐fronted Geese captured in Texas and Louisiana, from 7 days before the first migratory movement until incubation initiation, spanning 2016–2019.

To characterize migration routes and stopover areas, we used 62 complete migration tracks from 57 individual White‐fronted Geese (retaining 1 additional track from each of 5 individuals because migration routes and stopover areas can vary by year; Figure 1) to create a UD for each individual per year. We used dBBMM in the “move” package (Kranstauber et al., 2018) in program R (version 3.6.2, R Core Team, 2019) using a margin size = 9, a window size = 25, and a location error = 8 m, to develop UDs with a 10 km^2^ grid size. These parameters correspond to an overall temporal moving window of 24 h and allow for both intra‐ and inter‐day variation in identifying movement characteristics (Buechley et al., 2018; Kranstauber et al., 2012). We developed a UD for each individual by weighting each UD pixel by the total number of days spent within the pixel during migration. We then summed all individual UDs across years and normalized the final composite UD so that the sum of all pixel values was equal to 1 (Buechley et al., 2018; Palm et al., 2015; Sawyer et al., 2009). We calculated UD probability densities, or the relative frequency of occurrence of an animal in space and time (Keating & Cherry, 2009), at the 99%, 90%, 80%, 70%, 60%, and 50% levels to display a high‐resolution gradient of White‐fronted Goose space use throughout spring migration. This allowed us to identify areas of high use (50%) and moderate use (60%–80%), as well as the overall extent of migration space (99%; Buechley et al., 2018). We calculated the area (km^2^) of high and moderate use and the percent of the total area for each respective use category within each U.S. state and Canadian province in the Central and Mississippi Flyways (excluding Texas and Louisiana where devices were deployed). We did not calculate the use areas >60° N to avoid including areas where White‐fronted Geese spent time moving locally around nesting locations during the pre‐nesting period prior to incubation initiation.

**FIGURE 2:**
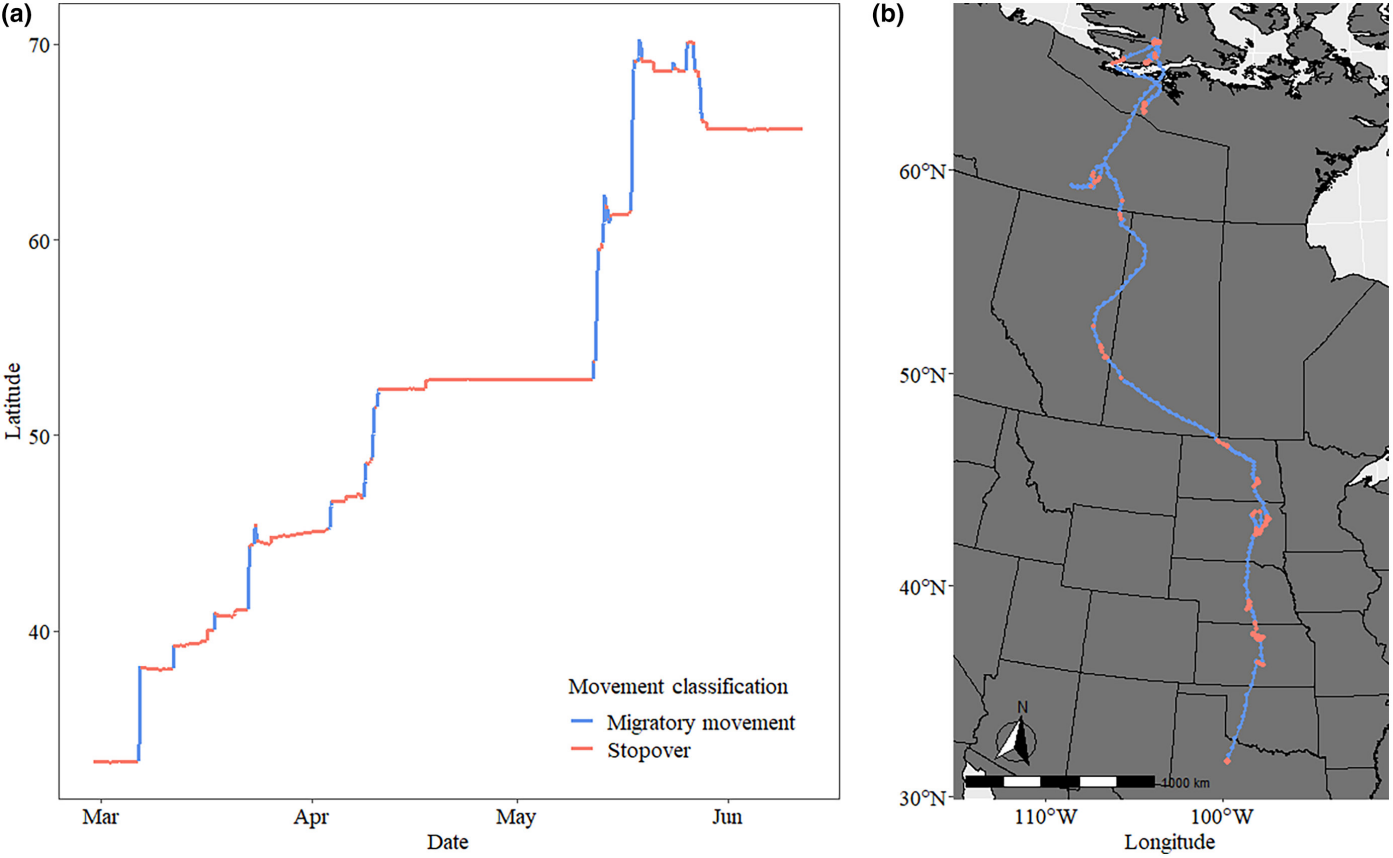
Spring migration movements of a Greater White‐fronted Goose displaying migratory flight movements and stopover periods (a) across latitudes through time and (b) spatially represented by global positioning system (GPS) tracks. Trajectory segmentation into migration and stopovers was conducted through hidden Markov models.

We began analyzing the movement trajectories on wintering areas 7 days before the first migratory movement northward (≥50 km) determined by calculating net‐squared displacement of the trajectory. As an endpoint for each trajectory, we used the incubation initiation date for nesting individuals or the median incubation initiation date per year for individuals that did not nest. Within that trajectory, we aimed to describe migration characteristics. Breeding propensity in geese can be influenced by the breeding status of the previous year, in which individuals who raised a brood in year ${\text{year}}_{t-1}$ may be less likely to breed in ${\text{year}}_{t}$, and vice‐versa (Reed et al., 2004; Souchay et al., 2014; Viallefont et al., 1995, but see Sedinger et al., 2008). Therefore, to reduce bias associated with breeding propensity, we removed one trajectory from five individuals that had complete data for two spring periods and retained the trajectory that resulted in a breeding attempt, or had higher quality GPS data (i.e., more GPS locations, less missing fixes) if a breeding attempt did not occur in either year. We removed one additional individual due to frequent gaps in data throughout the track. Therefore, we used a total of 56 individuals (50 females and 6 males, including no known pair‐bonded individuals) across 4 years. We used the “moveHMM” package (Michelot et al., 2016) to fit HMMs to the migration trajectories to distinguish long‐distance migration movements from stopover and local movements assigned directly to the GPS locations to segment the trajectory itself into movement categories. We decided a priori to fit 2‐, 3‐, and 4‐state HMMs with speed (adding 0.1 to each speed measurement at each location to avoid zero‐inflation, km/h), recorded from the tracking device as a covariate, as we believed there to likely be no more than four biologically interpretable states, including (1) roosting, (2) foraging, (3) local, short‐distance flights, and (4) long‐distance, migratory flights (Pohle et al., 2017). We used global state decoding and the Viterbi algorithm approach to determine the most likely state sequence (Leos‐Barajas & Michelot, 2018). We chose a four‐state model after visualizing movement trajectories, assessing resulting state classifications, and evaluating pseudo‐residuals of better fitting models, and concluded that it provided the most biologically meaningful interpretability of movements (Pohle et al., 2017). We then fit this model to each individual because of variation in migration and movement strategies among individuals. We were not interested in determining specific fine‐scale behaviors (e.g., foraging and roosting), but rather to segment the movement trajectory into large‐scale movements indicative of migratory movements between stopovers, and short, localized movements during stopovers, similar to a large‐scale version of “encamped” and “exploratory” movements (Edelhoff et al., 2016). Therefore, we interpreted the state with the largest mean step length and lowest mean turning angle as the “migratory movement” state (i.e., segments of the track with the longest and straightest segments, indicating fast, and directed travel; Pohle et al., 2017). We then combined all three remaining states with shorter mean step lengths and larger mean turning angles which were classified as “stopover” segments of the trajectory, except for the final stopover near breeding areas which started the pre‐incubation duration period. The HMMs resulted in an average step length for the migratory movements classification of 32.3 ± 0.35 km/step (*n* = 56). To ensure migrations were continuous and not the result of a single missing GPS location, we further restricted movement segments that were classified as migratory flight by requiring them to be at least twice the mean step length for this state (64.6 km). The resulting “migratory flight” step length is slightly larger than other step lengths used to define migratory movements in geese (i.e., 30–50 km, see Drent et al., 2007; Kölzsch et al., 2015). The final result was a complete migration track for each individual with segments classified into either a “migratory movement” or “stopover” state (Figure 2).

### 2.4 Migration characteristics

We calculated total migration distance as the sum of step lengths (km), and migration duration as the total number of days from the first migratory movement until the end of the last migratory movement, acknowledging that geese still make small‐scale, local movements while settling prior to nesting. The number of stopovers was the total count of segments classified as “stopover” for each individual determined from the HMM. Additionally, we summed the total number of reverse migratory movements (i.e., migratory movements that resulted in a net decrease in latitude using the same distance criteria as a migratory movement) to account for individuals that retreated from advancements during spring migration, likely due to inclement weather. We determined the last wintering region as the region of the last GPS location prior to the first migratory movement, classified as the MAV, Chenier Plain of Louisiana, Rolling/High Plains of Texas, or Other, where individuals resided outside of these regions (VonBank, 2020). We calculated the percentage of time each marked individual spent in high‐use migratory areas from the UD based on the number of GPS locations within high‐use areas divided by the total number of locations during migration, multiplied by 100. We also determined the arrival to breeding areas as the ordinal date of the end of the final migratory movement. For individuals who attempted a nest, we calculated the pre‐nesting duration as the number of days between migration end and incubation initiation. Finally, we investigated whether incubation initiation date influenced the probability of nesting success.

### 2.5 Breeding determination

**FIGURE 3:**
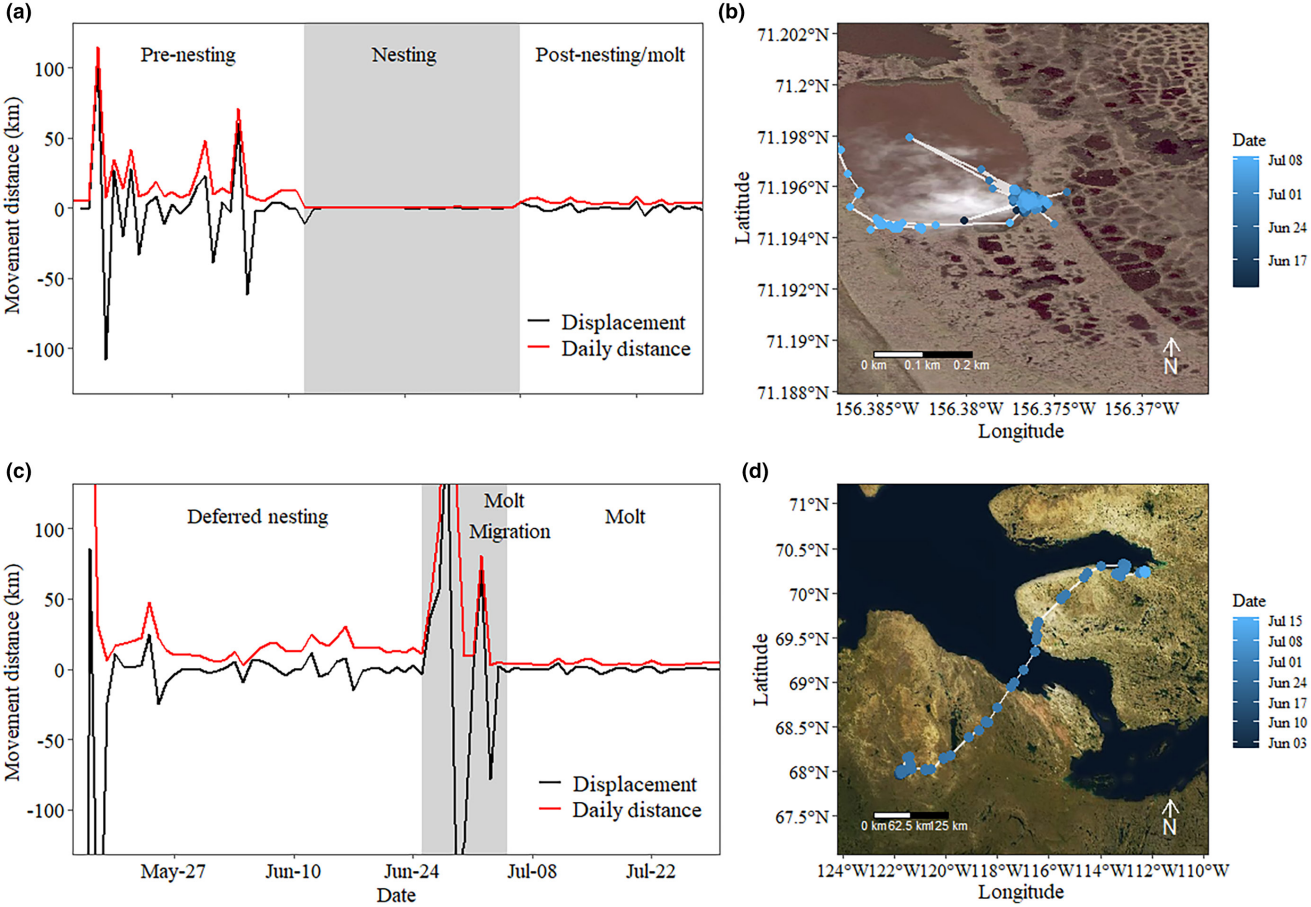
Example of daily distance traveled and displacement (km) from previous global positioning system (GPS) location for an individual Greater White‐fronted Goose that (a) attempted breeding showing clear pre‐nesting, nesting, and post‐nesting movements, or (c) deferred breeding in a given year, showing no periods of reduced movements. Breeding geese displayed (b) highly clustered GPS locations centered on nest or (d) continued moving locations throughout the breeding period, including making molt migrations. Photographic images from Esri via the R package mapview (Appelhans et al., 2023).

We used a combination of movement characteristics to determine whether an individual attempted or deferred breeding in a given year. Midcontinent White‐fronted Geese lay an average of 4.1 eggs/clutch (Ely & Dzubin, 1994), laying approximately 0.8 eggs/24 h (Baldassarre, 2014), and incubation begins following laying of the last egg. Therefore, we determined if an individual attempted to breed by a combination of minimal movement for ≥6 days and repeated locations at a single site. We were precluded from detecting individuals that failed nesting during the egg‐laying period prior to incubation, and only report breeding outcome statistics for individuals that successfully initiated incubation. Additionally, we did not attempt to determine failure during the brood‐rearing period, and all analyses were of characteristics prior to incubation or median incubation initiation date for non‐attempting individuals. We calculated total daily step length (km) and consecutive step displacement (distance from previous step; km) using all GPS locations from 15 May through 30 July each year. Total daily distances that totaled ≤2 km/day (Kölzsch et al., 2015) for ≥6 days indicated a nesting attempt. We also visually inspected location plots in program R to increase our confidence that a location with highly clustered GPS locations paired with a period of minimal movement indicated a nesting attempt (Figure 3). Further, we determined whether a nesting attempt either succeeded or failed based on duration of the signature of the nesting attempt. The incubation period of an average clutch is 23 days in the Canadian Arctic (Barry, 1967), which is a minimum estimate in published literature. Thus, we classified a nesting attempt as successful if the period with daily step lengths of ≤2 km lasted at least 23 days, and failed if less than 23 days. An individual that deferred nesting was apparent because it did not show an affinity to a single location for at least 6 days, did not exhibit daily step lengths of ≤2 km, and often conducted a molt migration during late June to early July (Figure 3).

### 2.6 Statistical analysis

We performed Pearson correlation analysis with all variables to determine levels of correlation for both breeding attempts and nest success and excluded highly correlated variables within the same model. We fit two separate mixed‐effects logistic regressions to model the effects of migration characteristics on breeding attempt or deferral, and for individuals that attempted breeding, on whether they were apparently successful or failed. We included year as a random effect with four levels (2016–2019) to account for year‐specific factors influencing migration characteristics and breeding outcomes. All migration characteristics were centered and standardized. We fit models using program R in the “lme4” package (Bates et al., 2015). We used Akaike's Information Criterion adjusted for small sample sizes (AICc, Burnham & Anderson, 2002) to evaluate, rank, and select the top‐ranked model (using ΔAICc and Akaike weights *w*_*i*_) from all possible model combinations of main effects and a priori chosen interactions using the “MuMIn” package (Barton, 2019). We used model averaging to obtain parameter estimates of competitive models (i.e., ≤6 ΔAICc), and report conditional model‐averaged parameter estimates (Grueber et al., 2011). We determined statistical significance at *p* ≤ .05. We considered the following migration characteristics to explain variation in breeding attempt or deferral: migration distance (km), migration duration (days), number of stopovers, number of reverse migratory movements, region where migration began from (using the MAV as the reference category), the proportion of time spent in high use stopover areas, and arrival date to breeding areas (ordinal date). To investigate factors influencing nest success or failure of geese that did attempt to nest, we included all variables above except for last wintering region (due to insufficient observations from each region) and included the ordinal date of incubation initiation and the duration of the pre‐nesting period.

To determine if there were differences in migration characteristics relative to the region where migration began, we used separate analysis of variance *F*‐tests in program R to test the null hypothesis that there was no difference in each migration characteristic among winter regions after assessing homogeneity of variances and residuals for normality using Shapiro‐Wilks test. We tested for differences in migration duration, total migration distance, number of stopovers, and number of reverse migratory movements among winter regions.

Finally, we detected potential temporal differences in arrival date, pre‐nesting duration, and incubation initiation dates in relation to breeding success between eastern and western portions of the breeding range. Because this correlation may mask the effects of migration characteristics on breeding outcomes, we classified White‐fronted Geese into two broad geographic range groups; Western breeders (i.e., Interior Alaska, West Coast/Seward Peninsula, and North Slope Alaska) and Eastern breeders (all Nunavut and Arctic Islands). We conducted mixed‐effects logistic regressions on breeding status in the same manner as at the population level for each geographic group. Due to small sample sizes for reproductive success and convergence issues with mixed‐effects models, we removed year as a random effect and used separate multiple logistic regressions for reproductive success for all years combined for each geographic range. We also limited the number of explanatory variables to those directly related to migration; migration duration, total migration distance, number of stopovers, and proportion of time spent in high‐use areas. We used separate two‐sided Mann–Whitney *U* tests (Mann & Whitney, 1947) for Eastern and Western breeders to determine if median arrival date and median incubation initiation date were different between successful and unsuccessful individuals within each region. Additionally, we examined the correlation between pre‐nesting duration and arrival date by categorizing observations by all breeding areas and reproductive outcomes to determine if breeding region influenced where individuals fell along a capital‐income continuum (Klaassen et al., 2006).

## 3 RESULTS

### 3.1 Migration routes and space use

**FIGURE 4:**
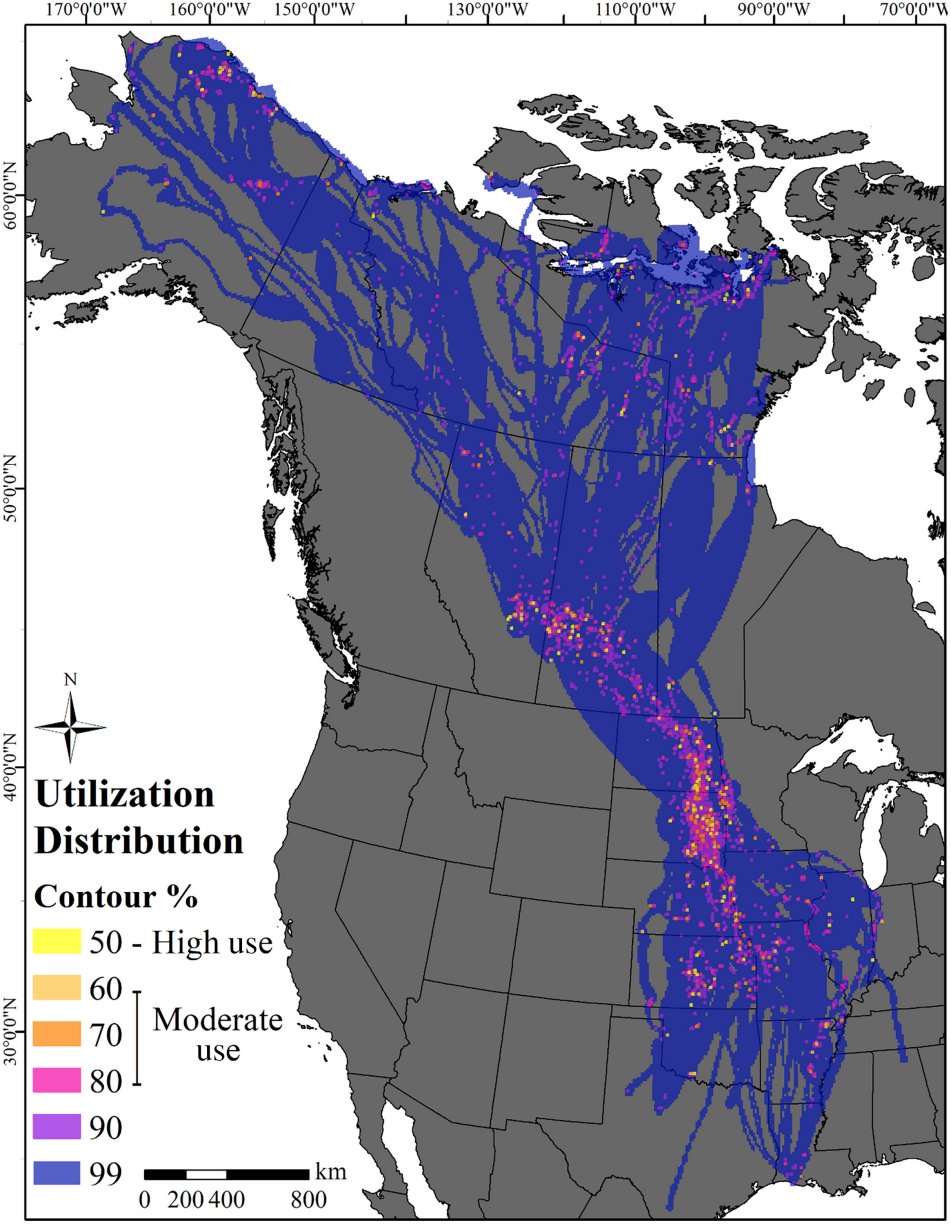
Cumulative dynamic Brownian bridge movement model for 57 Greater White‐fronted Geese captured in Texas and Louisiana during spring migrations, 2016–2019.

Spring migration of White‐fronted Geese covered large portions of Midcontinent North America. Portions of migration tracks through the Great Plains region of the United States and Canada were highly concentrated, while other portions were much more segregated, such as tracks from Alberta and Saskatchewan crossing the boreal forest to Arctic or subarctic breeding locations (Figure 1). A total of 128,900 km^2^ of migration space was classified as high‐use (9000 km^2^) and moderate‐use (119,900 km^2^) combined across 4 years of spring migration (Figure 4, Table 1). Saskatchewan provided the greatest amount of high‐use area (13.3% of total high‐use area), followed by South Dakota (12.9%), North Dakota (12.2%), and Kansas (10.0%) with other states and provinces providing <10.0% of the total high‐use area (Table 1). South Dakota provided the greatest amount of moderate‐use area (13.4% of total moderate‐use area), followed by North Dakota (7.6%), Saskatchewan (6.7%), and Kansas (4.2%) with other states and provinces providing <4.0% of the total moderate‐use area (Table 1). Combining high‐use and moderate‐use areas, South Dakota was ranked first in total use area combined during spring migration (13.3%), followed by North Dakota (7.9%), Saskatchewan (7.2%), Kansas (4.7%), and Alberta (3.8%) (Table 1).

**TABLE 1:** Area (ha), percent of total area, and rank of high‐use (50%), moderate‐use (60%–80%), and combined high‐ and moderate‐use areas calculated from a utilization distribution derived from a dynamic Brownian bridge movement model of 57 Greater White‐fronted Geese captured in Texas and Louisiana during spring migrations, 2016–2019, of for each U.S. State or Canadian Province (south of 60° N latitude).

Region	State/province	High‐use			Moderate‐use			Combined		
		Area^a^	%	Rank^b^	Area	%	Rank	Area	% Of total	Overall rank
Central Flyway	Alberta	700.0	7.8	5	4172.2	3.5	5	4872.2	3.8	5
	Saskatchewan	1200.0	13.3	1	8061.6	6.7	3	9261.6	7.2	3
	North Dakota	1100.0	12.2	3	9064.6	7.6	2	10,164.6	7.9	2
	South Dakota	1161.5	12.9	2	16,033.4	13.4	1	17,194.9	13.3	1
	Nebraska	418.5	4.7	6	2872.2	2.4	6	3290.7	2.6	6
	Kansas	900.0	10.0	4	5095.5	4.2	4	5995.5	4.7	4
	Oklahoma	1000.0	1.1	11	600.0	0.5	13	700.0	0.5	13
Mississippi Flyway	Manitoba	3000.0	3.3	8	1068.5	0.9	12	1368.5	1.1	11
	Minnesota	338.5	3.8	7	2625.3	2.2	8	2963.8	2.3	7
	Iowa	281.5	3.1	10	2530.4	2.1	9	2811.9	2.2	8
	Illinois	3000.0	3.3	8	1152.7	1.0	11	1452.7	1.1	10
	Indiana	0	0.0	12	400.0	0.3	14	400.0	0.3	14
	Missouri	0	0.0	12	2722.1	2.3	7	2722.1	2.1	9
	Arkansas	0	0.0	12	1200.0	1.0	10	1200.0	0.9	12
Total	–	9000.0	–	–	119,900.0	–	–	128,900.0	–	–

^a^ Area measured in km^2^.

^b^ Rank based on largest amount of area per category individually ranked for high‐use, moderate‐use, and combined.

### 3.2 Migration characteristics

White‐fronted Geese spent an average of 92.7 ± 1.9 (SE) days from migration initiation until arrival on breeding areas (range = 65–123 days) across years, and 108.2 ± 2.3 days (range = 82–130 days) from migration initiation until incubation initiation for individuals that attempted nesting. On average, White‐fronted Geese traveled a cumulative migration distance of 5452 ± 124 km (range = 3884–7690 km), made 15.7 ± 0.6 number of stopovers (range = 7–33), and 0.9 ± 0.2 number of reverse migratory movements (range = 0–4) during spring migration. The number of stopovers and number of reverse migratory movements were moderately correlated (*r* = .61), and migration duration was positively correlated with arrival date (*r* = .80). For individuals that attempted breeding, migration duration, and incubation initiation date were positively correlated (*r* = .76). Further, arrival date was positively correlated with migration duration (*r* = .83) and negatively correlated with pre‐nesting duration (*r* = −.67), and arrival date was removed from all models of reproductive success, except in a univariate model. We found no evidence that any of the observed migration characteristics were different among White‐fronted Geese originating from different winter regions, including total migration distance (*F*_3,52_ = 0.31, *p*‐value = .82), total migration duration (*F*_3,52_ = 0.32, *p*‐value = .810), number of stopovers (*F*_3,52_ = 0.87, *p*‐value = .46), or number of reverse migratory movements (*F*_3,52_ = 1.22, *p*‐value = .31; Table 2).

**TABLE 2:** Mean and standard error of migration characteristics from Greater White‐fronted Geese (*n* = 56) beginning migration in distinct wintering regions in North America, during spring migration 2016–2019.

Winter region	*n*	Migration characteristics			
		Total distance (km)	Duration (days)	# Stopovers	# Reverse migratory movements
Mississippi Alluvial Valley	5	5070.2 ± 394.3	97.0 ± 7.8	15.8 ± 1.8	1.4 ± 0.4
Chenier plain	24	5514.2 ± 162.9	93.8 ± 3.0	16.3 ± 0.6	0.6 ± 0.2
Rolling/high plains	8	5465.2 ± 258.2	91.8 ± 5.2	16.5 ± 1.3	1.2 ± 0.3
Other	19	5472.0 ± 332.2	90.6 ± 3.3	18.9 ± 1.2	0.9 ± 0.4

### 3.3 Breeding status

We determined that 55.4% of all individuals attempted to breed, while 44.6% deferred (or attempted but failed prior to incubation as we could not detect these attempts), but the percentage that attempted was variable among 2016 (40.0%, *n* = 5), 2017 (87.5%, *n* = 8), 2018 (37.5%, *n* = 8), and 2019 (54.3%, *n* = 35). The proportion of breeding attempts by region was 75.0% in Eastern Nunavut (*n* = 8) and Western Alaskan Coast/Seward Peninsula (*n* = 4), 58.8% on the Alaskan North Slope (*n* = 17), 50% in Central Nunavut (*n* = 10), 54.5% in Western Nunavut/Arctic Islands (*n* = 11), and 33.3% in Interior Alaska (*n* = 3), while the Northwest Territories had no breeding attempts (0.0%, *n* = 3). Although we did not include two breeding seasons for the same individual in analyses, five White‐fronted Geese provided complete data for two breeding periods. Two individuals attempted nesting in both years, two individuals attempted only 1 year, and one individual did not attempt in either year. Individuals who attempted in both years were also successful both years. Individuals attempting to breed arrived on breeding areas on average 2 days (±2.3 days SE) earlier than deferring individuals, and they spent 15.6 ± 1.2 days (range = 4–28 days, *n* = 31) in the pre‐nesting period. Median incubation initiation date varied by only 7 days across years overall breeding areas and was earliest in 2017 (07 June), intermediate in 2018 (12 June) and 2019 (12 June), and latest in 2016 (14 June). We determined that 71.0% of all individualswho attempted to breed were successful (*n* = 22), and 29% failed (*n* = 9). Nest success was generally high across years, but was subject to small sample sizes in some years. Nest success was 100% in 2016 (*n* = 2), 57.1% in 2017 (*n* = 7), 100% in 2018 (*n* = 3), and 68.4% in 2019 (*n* = 19). Failed individuals spent an average of 14.1 ± 1.29 days incubating prior to failure, while successful breeders spent 26.8 ± 0.34 days incubating on average. In the western region, 51.9% of individuals (*n* = 27) attempted to nest and 57.1% of those were successful. In the eastern region, 58.6% of all individuals (*n* = 29) attempted to nest, and 82.4% of those were successful. White‐fronted Geese breeding in the western portion of the breeding range arrived 18 days earlier (134 median ordinal date), had a 90.9% longer pre‐nesting period, and initiated nests 9 days earlier (158 median ordinal date) than White‐fronted Geese in the east.

### 3.4 Effects of migration characteristics on breeding outcomes

After model averaging, the final model explaining variation in breeding attempt/deferral at the population level included arrival date, proportion of migration spent in high‐use areas, total migration distance, migration duration, number of stopovers, number of reverse migratory movements, and winter region; however, all coefficients had confidence intervals (CIs) that overlapped zero, indicating none were significant predictors of breeding attempt/deferral (Table 3). The final model examining factors influencing nest success at the population level included proportion of migration spent in high‐use areas, number of stopovers, number of reverse migratory movements, total migration distance, migration duration, pre‐nesting duration, and incubation initiation. All coefficients had CIs that overlapped zero and were not strong predictors of breeding outcomes at the population level (Table 3). For breeding status and reproductive success in western and eastern breeding regions, respectively, none of the individual variables were statistically significant in any of the final model‐averaged models (Table 3, Table S1).

**TABLE 3:** Final model‐averaged models of migration and nesting characteristics influencing reproductive outcomes, *β*‐coefficients, and 95% confidence intervals for Greater White‐fronted Geese at the population, eastern‐, and western‐breeding levels in North America, 2016–2019.

Reproductive outcomes	Analysis level	Model‐averaged terms	*β*	95% confidence interval
Attempt/deferral	Population	Arrival date	−.31	−1.07 to 0.46
		Proportion in high‐use area	−.19	−0.75 to 0.36
		Total migration distance	−.08	−0.66 to 0.51
		Migration duration	.16	−0.68 to 1.00
		Number of stopovers	.001	−0.56 to 0.56
		Reverse migratory movements	−.06	−0.61 to 0.50
		Winter region—Chenier Plain	.91	−1.10 to 2.93
		Winter region—Rolling Plains	.41	−1.91 to 2.72
		Winter region—Other	.51	−1.54 to 2.56
	East breeding	Arrival date	.14	−1.25 to 1.53
		Proportion in high‐use area	.15	−0.74 to 1.04
		Total migration distance	.12	−1.09 to 1.33
		Migration duration	.89	−0.32 to 2.10
		Number of stopovers	.12	−1.12 to 1.35
		Reverse movements	−.08	−0.92 to 0.76
	West breeding	Arrival date	−2.73	−5.73 to 0.25
		Proportion in high‐use area	−.36	−1.65 to 0.93
		Total migration distance	.46	−0.75 to 1.67
		Migration duration	.49	−1.11 to 2.09
		Number of stopovers	.17	−0.83 to 1.18
		Reverse migratory movements	−.05	−1.28 to 1.17
Success/failure	Population	Proportion in high‐use area	1.27	−0.63 to 3.16
		Number of stopovers	−.59	−1.99 to 0.80
		Reverse migratory movements	.28	−0.79 to 1.35
		Total migration distance	.44	−1.18 to 2.06
		Migration duration	.16	−0.79 to 1.11
		Pre‐nesting duration	−.62	−1.77 to 0.52
		Incubation initiation	−.44	−1.40 to 0.52
	East breeding	Migration duration	−1.77	−4.37 to 0.83
		Total migration distance	.25	−1.69 to 2.20
		Proportion in high‐use area	.65	−1.79 to 3.08
		Number of stopovers	−1.02	−3.98 to 1.93
	West breeding	Migration duration	.47	−1.53 to 2.48
		Total migration distance	1.15	−1.80 to 4.09
		Proportion in high‐use area	1.08	−1.85 to 4.00
		Number of stopovers	−.79	−2.86 to 1.28

**FIGURE 5:**
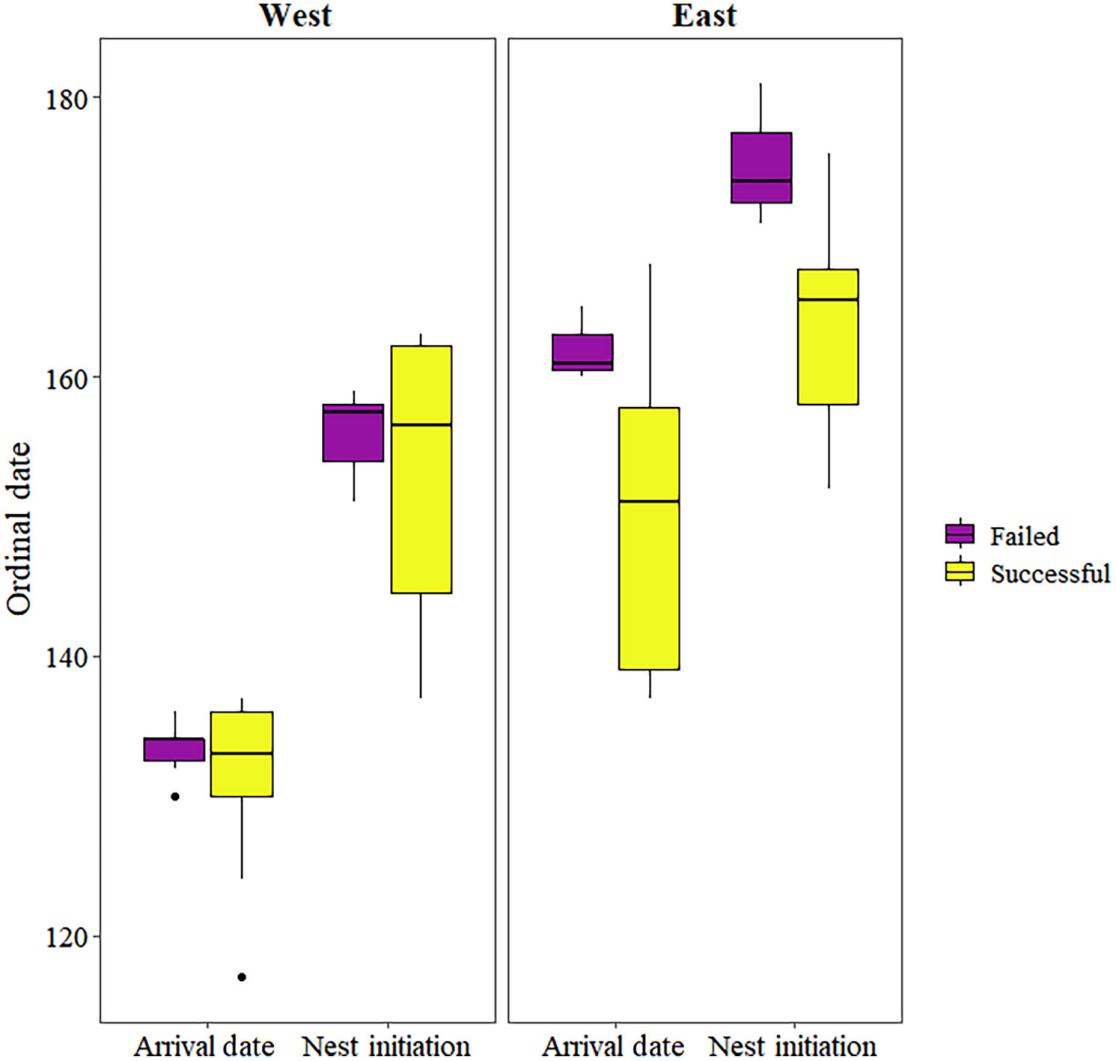
Median arrival dates and incubation initiation dates between successful and failed Greater White‐fronted Geese for eastern and western geographic breeding regions in North America during spring 2016–2019.

**FIGURE 6:**
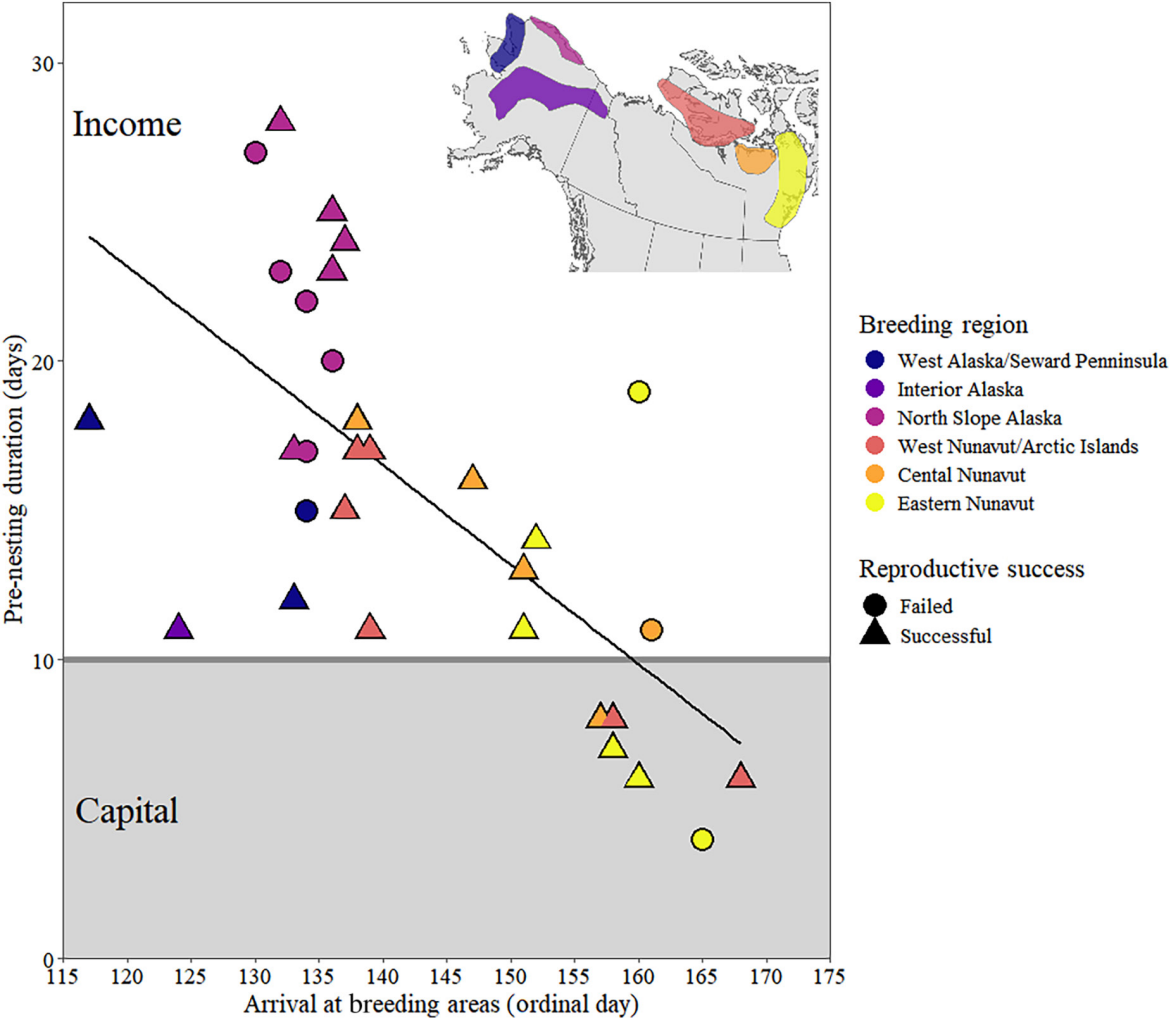
Pre‐nesting duration as a function of arrival to breeding areas (ordinal date) across six breeding areas of Greater White‐fronted Geese, 2016–2019. Separation of the figure into “income” and “capital” breeding strategies based on <10 days pre‐nesting duration required for use of endogenous reserves for egg production (Klaassen et al., 2006). Inset map of breeding areas corresponding to individuals defined by VonBank et al. (2021) and figure style influenced by Klaassen et al. (2006). Declining trendline indicates transition from income to capital strategy as pre‐nesting duration decreases and arrival date increases.

We detected differences in the relationship between reproductive success and arrival date for western and eastern breeding areas. Within western breeders, there was no difference in median arrival date (*W* = 25.5, *p*‐value = .90), pre‐nesting duration (*W* = 24.0, *p*‐value = 1.00, range of failed breeders = 17–29 days, range of successful breeders = 13–30 days), or incubation initiation date (*W* = 23.0, *p*‐value = .95) between successful and failed breeders, where the difference in median arrival days was only 1 day, pre‐nesting duration less than 1 day, and incubation initiation only 2 days (Figure 5). However, in eastern breeders, the median arrival date of successful breeders was 10 days earlier (*W* = 38.5, *p*‐value = .03) and incubation initiation date was 8 days earlier (*W* = 40.0, *p*‐value = .02) than failed breeders (Figure 5). We found no difference in pre‐nesting duration between successful and failed breeders (*W* = 20, *p*‐value = .95, range of failed breeders = 6–21 days, range of successful breeders = 8–20 days) for eastern breeders. We found a strong longitudinal gradient in correlation between arrival date and pre‐nesting duration (Figure 6), indicating western geese invoke a more income‐based breeding strategy, while eastern geese use a more mixed capital/income‐based breeding strategy (Klaassen et al., 2006).

## 4 DISCUSSION

We found no evidence that spring migration characteristics prior to arrival to breeding areas influenced Midcontinent White‐fronted Goose breeding outcomes at the population level, or within eastern and western breeding regions. It appears that factors upon arrival to breeding areas (e.g., snow and ice cover, timing of vegetation growth, and predation), may play a larger role in breeding outcomes than factors during migration (Legagneux et al., 2012). Bêty et al. (2004) found that female greater Snow Geese (*A. caerulescens atlanticus*) showed heterogeneity in migration characteristics among individuals (e.g., migration duration and arrival date), but individuals themselves were highly consistent among years, suggesting there is flexibility in migration strategies that result in successful breeding outcomes, potentially through the mechanism of individual quality. Weegman et al. (2017) found that time‐ and energy‐budgets for Greenland White‐fronted Geese (*A.a. flavirostris*) were similar between breeders and non‐breeders throughout spring migration and summer, suggesting that factors affecting breeding deferral occurred on breeding areas or as a result of previous breeding effort and were not the result of migration characteristics. This is further supported by no detectable effects of the proportion of time spent feeding during specific segments of spring migration on breeding outcomes in White‐fronted Geese, although greater overall energy expenditure and lower proportion of time spent feeding during the entirety of migration was associated with increased rates of breeding deferral (Cunningham et al., 2022).

Space‐use during spring migration was previously unquantified for Midcontinent White‐fronted Geese. Because wintering region where migration was initiated had no effect on breeding outcomes, we found no evidence of a trade‐off between greater energy expenditure and increased time spent feeding in contemporary wintering regions cascading through spring migration and reproductive success (VonBank et al., 2021, 2023). Additionally, the proportion of time spent in high‐use areas during spring migration did not influence breeding outcomes. We hypothesized that areas of high use were indicative of high‐quality resources (Fretwell & Lucas, 1969), and individuals who spent more time in those areas would be more likely to breed and be successful. Geese spent the majority of spring migration in South Dakota, North Dakota, and Saskatchewan, which are agriculturally rich areas that provide substantial energy for spring migrating geese (Alisauskas, 2002; Pearse et al., 2011). The Midcontinent landscape allows geese to exploit agricultural waste grains continuously from wintering areas in the southern United States to staging areas in prairie Canada, which is roughly one‐half to two‐thirds of the total migration distance depending on winter origin and breeding region. White‐fronted Geese and lesser Snow Geese continually increase lipid storage as migration progresses northward through the Midcontinent region due to abundant agriculture, suggesting that foraging on waste grains can meet the energy demands of spring migration for these species (Alisauskas, 2002; Fowler et al., 2020; Krapu et al., 1995, 2004; Pearse et al., 2011). Furthermore, we could not detect a difference in breeding attempts or success based on the number of stopovers, or the number of reverse migratory movements (which undoubtedly increases energy expenditure). Use of high‐energy waste grains throughout migration likely mitigates the effect of increased energy expenditure associated with a greater number of migratory movements and reverse migratory movements.

While we did not calculate the use of areas north of 60° N in our migratory UD, our dBBMM indicated that White‐fronted Geese make subarctic stopovers prior to arriving to nesting locations, particularly in eastern Northwest Territories and Nunavut. Subarctic stopovers are necessary to consume natural foods with higher protein content than agricultural grains, where individuals can continue to build endogenous reserves prior to arrival to breeding locations (Alisauskas & Ankney, 1992; Bêty et al., 2003; Fox & Abraham, 2017). In the western portion of the breeding range, several White‐fronted Geese used the Yukon Flats National Wildlife Refuge as a stopover area en route to North Slope and western Alaska breeding areas, but individuals breeding in western breeding areas did not use subarctic stopovers as frequently or as broadly as birds breeding in eastern portions of the breeding range (Figure 4). Spatial variation in use, environmental conditions, and foraging efficiency in subarctic staging areas across the geographic range of White‐fronted Geese may contribute to observed differences in timing of arrival and pre‐nesting duration among eastern and western regions (Bêty et al., 2004). Future research could examine the contributions of staging ecology to breeding outcomes.

We found evidence that Midcontinent White‐fronted Geese employ variable breeding strategies between eastern and western breeding areas. Western‐breeding White‐fronted Geese arrived earlier, had longer pre‐nesting durations, and initiated nests earlier than eastern breeders. However, among western breeders there was no difference in these variables between failed and successfully nesting individuals. Contrastingly, for eastern breeders, successfully reproducing individuals arrived and initiated nests earlier than those that failed. Additionally, we found longitudinal differences in the relationship between arrival date and pre‐nesting duration across breeding areas, which may be due to environmental variation across the North American Arctic. During 1967–2004 at latitudes between 60 and 70° N, mean date of snow melt showed a west to east gradient, and was approximately 20 days earlier in the western Arctic (i.e., North Slope) than the eastern Arctic (Nunavut; Foster et al., 2008), which closely matches with western‐breeding White‐fronts arriving 18 days earlier than eastern breeders. Klaassen et al. (2006) suggested that a pre‐nesting duration of <10 days requires eggs to be partially developed from endogenous reserves, as rapid follicle maturation takes approximately that long in geese (Alisauskas & Ankney, 1992). In our study, none of the geese in the western Arctic and 35% of the geese in the eastern Arctic had pre‐nesting periods of <10 days. Thus, western breeders showed a likelihood for an income‐based strategy, and eastern breeders tended towards a more capital‐based strategy.

Breeding propensity was similar between eastern and western White‐fronted Goose breeders, but nest success was greater in eastern breeders than western breeders. Alisauskas (2002) showed that vanguard flocks of lesser Snow Geese (*A.c. caerulescens*) arriving to breeding areas were fatter than those at more southerly areas on the same date, suggesting early arrivers may need greater endogenous body stores to accommodate limited food resources in early spring and then use exogenous reserves to complete follicle development. In eastern portions of the breeding range, White‐fronted Geese appear to arrive later and have a shorter pre‐nesting period. Eastern White‐fronted Geese are likely required to rely more on endogenous reserves for follicle development as their time to forage on breeding areas is constrained by nest initiation timing and lack of vegetation growth, limiting their ability to sequester nutrients and energy on breeding areas to support follicle development (Klaassen et al., 2006; Lameris et al., 2018). Eastern‐breeding White‐fronted Geese took more time at subarctic stopovers where they can likely deposit greater endogenous reserves that can be incorporated into eggs, compared to western‐breeding geese that arrive earlier in the spring with longer pre‐nesting durations and fewer subarctic stopovers. The observed variability in arrival date and pre‐nesting duration between eastern and western breeding regions and potential reliance on endogenous reserves may explain why we could not detect the effects of migration characteristics on breeding status at the population level. Body condition on arrival may play a large role in pre‐nesting duration across regions, as geese that arrive early with smaller endogenous stores likely require longer pre‐nesting durations than geese that arrive later (Bêty et al., 2003; Prop et al., 2003; Tombre et al., 1996). Additionally, predation rates, predator community composition, or local weather events may differentially influence nest success between regions. Due to the expansive breeding range of White‐fronted Geese, previous studies investigating migration, pre‐breeding, and nesting biology have been spatially limited, usually restricted to a single geographic region (Budeau et al., 1991; Hupp et al., 2018; Krapu et al., 1995); however, our results suggest that range‐wide variation in migration, body condition, and breeding strategies should be considered.

White‐fronted Geese are typically the first goose species to arrive to northern breeding areas in Midcontinent North America (Ely & Raveling, 1984; Hupp et al., 2018). Early arrival, typically prior to snow melt, and early nesting are advantageous to Arctic‐nesting species due to the short temporal window in which geese must complete nesting, molt, and allow gosling growth prior to fall migration, all while attempting to time the gosling growth period to match peaks in vegetation growth rate and nutrient levels (Lameris et al., 2018; MacInnes et al., 1974; Sedinger & Raveling, 1986). White‐fronted Geese experienced breeding success across the capital–income continuum, likely more so than species that experience greater time constraints between arrival and nest initiation (e.g., Black Brant [*Branta bernicla nigricans*], lesser Snow Goose, Hupp et al., 2018; Klaassen et al., 2006) and those that may not exhibit flexible enough strategies to include the use of exogenous reserves for follicle development in years of earlier spring phenology. Therefore, pre‐nesting duration should be important as to whether or not an individual attempts to nest, unless White‐fronted Geese deposit larger endogenous reserves into eggs to allow for shorter pre‐nesting duration (Drent & Daan, 1980). Budeau et al. (1991) reported that White‐fronted Geese on the Yukon‐Kuskokwim Delta of Alaska arrived 14–21 days before nest initiation, while Hupp et al. (2018) reported 24 and 28 days for two individuals on the Alaskan North Slope. However, we observed a wider range of pre‐nesting duration than previously reported, which further varied by region and did not differ between successful or failed individuals. Eastern breeders that were successful arrived and initiated nests earlier than those that failed, but pre‐nesting durations were similar. The effects of phenological mismatch between peak food quality and peak demand by young shorebirds have been shown to be stronger in the eastern Canadian Arctic than in the western Arctic (Kwon et al., 2019) and may explain the increased breeding success effects of early arrival and nest initiation that we observed in the eastern Arctic. Conversely, western breeders that failed arrived and initiated nests at the same time as those that succeeded. Therefore, breeding success may not be dependent on how much pre‐nesting time individuals have, but on how they use that time to mitigate possible lipid or protein deficiencies in addition to external factors (e.g., predation and extreme weather events) not related to individual quality or decision‐making.

Variation in migration and breeding strategies between eastern and western‐breeding White‐fronted Geese apparently allows multiple strategies to result in successful reproduction in a given year. High variability among migration characteristics and where individuals lay on the income‐capital breeding continuum varies by breeding region, further obscuring influences on breeding status at the population level. We contend that factors encountered on sub‐Arctic staging and on breeding areas likely dictate resulting breeding status for some portions of the population, and that plasticity in migratory strategy combined with high‐quality resources throughout much of migration may negate effects of variable migration strategies on breeding status, at least through the final stopover. Although we did not detect effects of migration characteristics on breeding outcomes, if migration costs are mitigated by high‐quality resources throughout migration, we may observe effects on breeding outcomes if such resources change in the future. While high‐quality resources such as agricultural waste grains may currently obscure migration effects, changing farming practices, climate‐driven changes to crop types or rotations, or anthropogenic‐driven farming needs (e.g., biofuels) may alter the ability of migrants to acquire necessary energy for successful migration and reproduction. While we did not include environmental effects (e.g., snow cover, precipitation rates, temperature), behavior (e.g., time spent foraging prior to nesting), or predation events, it is likely that these factors play a greater role in breeding outcome than do migratory characteristics (Cunningham et al., 2022).

## AUTHOR CONTRIBUTIONS

**Jay A. VonBank:** Conceptualization (equal); data curation (equal); formal analysis (lead); investigation (equal); methodology (lead); writing – original draft (lead); writing – review and editing (equal). **Kevin J. Kraai:** Conceptualization (equal); funding acquisition (equal); investigation (equal); methodology (equal); project administration (equal); writing – review and editing (equal). **Daniel P. Collins:** Conceptualization (equal); funding acquisition (supporting); investigation (equal); methodology (equal); resources (equal); writing – review and editing (equal). **Paul T. Link:** Data curation (equal); funding acquisition (equal); investigation (equal); methodology (equal); writing – review and editing (equal). **Mitch D. Weegman:** Formal analysis (supporting); investigation (equal); methodology (equal); writing – review and editing (equal). **Lei Cao:** Funding acquisition (equal); writing – review and editing (equal). **Bart M. Ballard:** Conceptualization (equal); funding acquisition (equal); investigation (equal); methodology (equal); project administration (equal); supervision (lead); writing – review and editing (equal).

## CONFLICT OF INTEREST STATEMENT

The authors declare no competing interest.

## Supporting information


Table S1.

## Data Availability

Data have been deposited in the Dryad Digital Repository for Peer‐Review, the Reviewer link is: https://datadryad.org/stash/share/fQBX6s9YhW6RXXJ8MPSCbBzGsKvKSEXSqrBHDQ_06VA. Data will be released upon publication of the manuscript.
