# Supplementary material for: Evidence of longitudinal differences in spring migration strategies of an Arctic‐nesting goose
Source: Ecol Evol. 2024 Sep 2;14(9):e11665. doi: 10.1002/ece3.11665 (PMC11368494; doi:10.1002/ece3.11665)
Supplement: Supplementary file 1 — Table S1. [file ECE3-14-e11665-s001.docx]

Supplementary Materials

Table S1. Generalized mixed-effects linear models included in model averaging examining factors influencing nesting attempt or deferral and nesting success or failure, from Midcontinent Greater White-fronted Geese, 2016-2019. Table includes response variable, model rank, variables included in each model, number of parameters (K), log likelihood (logLik), Akaike’s Information Criterion corrected for small sample size (AICc), difference in AICc from the top model (ΔAICc), and model weight (*w_i_*).

| Level | Response | Model | K | logLik | AICc | ΔAICc | *w_i_* |
| --- | --- | --- | --- | --- | --- | --- | --- |
| Population | Breeding Attempt or Deferral |  |  |  |  |  |  |
|  | 1 | NULL | 2 | -37.49 | 81.21 | 0.00 | 0.18 |
|  | 2 | Arrival Date | 3 | -38.24 | 82.94 | 1.73 | 0.08 |
|  | 3 | Proportion High-Use | 3 | -38.27 | 82.99 | 1.78 | 0.08 |
|  | 4 | Reverse Migratory Movements | 3 | -38.48 | 83.42 | 2.21 | 0.06 |
|  | 5 | Migration Distance | 3 | -38.48 | 83.43 | 2.22 | 0.06 |
|  | 6 | Migration Days | 3 | -38.49 | 83.45 | 2.24 | 0.06 |
|  | 7 | Stopovers | 3 | -38.49 | 83.45 | 2.24 | 0.06 |
|  | 8 | Arrival Date + Migration Days | 4 | -37.81 | 84.40 | 3.19 | 0.04 |
|  | 9 | Arrival Date + Proportion High-Use | 4 | -37.99 | 84.76 | 3.55 | 0.03 |
|  | 10 | Migration Distance + Arrival Date | 4 | -38.15 | 85.09 | 3.88 | 0.03 |
|  | 11 | Migration Distance + Proportion High-Use | 4 | -38.21 | 85.20 | 3.99 | 0.02 |
|  | 12 | Reverse Migratory Movements + Proportion High-Use | 4 | -38.22 | 85.22 | 4.01 | 0.02 |
|  | 13 | Arrival Date + Reverse Migratory Movements | 4 | -38.23 | 85.25 | 4.04 | 0.02 |
|  | 14 | Arrival Date + Stopovers | 4 | -38.24 | 85.26 | 4.05 | 0.02 |
|  | 15 | Proportion High-Use + Migration Days | 4 | -38.26 | 85.31 | 4.10 | 0.02 |
|  | 16 | Stopovers + Proportion High-Use | 4 | -38.27 | 85.31 | 4.10 | 0.02 |
|  | 17 | Migration Distance + Reverse Migratory Movements | 4 | -38.47 | 85.73 | 4.52 | 0.02 |
|  | 18 | Reverse Migratory Movements + Migration Days | 4 | -38.48 | 85.74 | 4.53 | 0.02 |
|  | 19 | Migration Distance + Stopovers | 4 | -38.48 | 85.74 | 4.53 | 0.02 |
|  | 20 | Migration Distance + Migration Days | 4 | -38.48 | 85.75 | 4.54 | 0.02 |
|  | 21 | Stopovers + Migration Days | 4 | -38.49 | 85.77 | 4.56 | 0.02 |
|  | 22 | Arrival Date + Proportion High-Use + Migration Days | 5 | -37.60 | 86.40 | 5.19 | 0.01 |
|  | 23 | Migration Distance + Arrival Date + Migration Days | 5 | -37.70 | 86.59 | 5.38 | 0.01 |
|  | 24 | Migration Distance + Arrival Date + Proportion High-Use | 5 | -37.77 | 86.74 | 5.53 | 0.01 |
|  | 25 | Arrival Date + Reverse Migratory Movements + Migration Days | 5 | -37.77 | 86.74 | 5.53 | 0.01 |
|  | 26 | Arrival Date + Stopovers + Migration Days | 5 | -37.80 | 86.79 | 5.58 | 0.01 |
|  | 27 | Starting Region | 5 | -37.93 | 87.06 | 5.85 | 0.01 |
|  | 28 | Arrival Date + Reverse Migratory Movements + Proportion High-Use | 5 | -37.95 | 87.10 | 5.89 | 0.01 |
|  | 29 | Arrival Date + Stopovers + Proportion High-Use | 5 | -37.99 | 87.17 | 5.96 | 0.01 |
|  | Breeding Success or Failure |  |  |  |  |  |  |
|  | 1 | Proportion High-Use | 3 | -17.39 | 41.70 | 0.00 | 0.09 |
|  | 2 | NULL | 2 | -18.68 | 41.82 | 0.12 | 0.08 |
|  | 3 | Pre-nesting Duration | 3 | -18.06 | 43.05 | 1.35 | 0.04 |
|  | 4 | Stopovers | 3 | -18.18 | 43.28 | 1.58 | 0.04 |
|  | 5 | Pre-nesting Duration + Proportion High-Use | 4 | -16.87 | 43.31 | 1.61 | 0.04 |
|  | 6 | Nest Initiation + Proportion High-Use | 4 | -16.88 | 43.34 | 1.64 | 0.03 |
|  | 7 | Reverse Migratory Movements + Proportion High-Use | 4 | -17.06 | 43.69 | 1.99 | 0.03 |
|  | 8 | Nest Initiation | 3 | -18.39 | 43.70 | 2.00 | 0.03 |
|  | 9 | Migration Distance + Proportion High-Use | 4 | -17.25 | 44.07 | 2.37 | 0.03 |
|  | 10 | Migration Days | 3 | -18.57 | 44.07 | 2.37 | 0.03 |
|  | 11 | Migration Distance | 3 | -18.61 | 44.15 | 2.45 | 0.03 |
|  | 12 | Pre-nesting Duration + Migration Distance + Proportion High-Use | 5 | -15.89 | 44.21 | 2.51 | 0.03 |
|  | 13 | Proportion High-Use + Migration Days | 4 | -17.32 | 44.21 | 2.51 | 0.03 |
|  | 14 | Stopovers + Proportion High-Use | 4 | -17.32 | 44.21 | 2.51 | 0.02 |
|  | 15 | Reverse Migratory Movements | 3 | -18.67 | 44.28 | 2.58 | 0.02 |
|  | 16 | Pre-nesting Duration + Stopovers | 4 | -17.45 | 44.48 | 2.78 | 0.02 |
|  | 17 | Pre-nesting Duration + Nest Initiation + Proportion High-Use | 5 | -16.06 | 44.56 | 2.86 | 0.02 |
|  | 18 | Pre-nesting Duration +Nest Initiation | 4 | -17.51 | 44.60 | 2.90 | 0.02 |
|  | 19 | Pre-nesting Duration + Arrival Date + Stopovers + Proportion High-Use | 6 | -14.70 | 44.94 | 3.24 | 0.01 |
|  | 20 | Nest Initiation + Stopovers | 4 | -17.83 | 45.24 | 3.54 | 0.01 |
|  | 21 | Nest Initiation + Reverse Migratory Movements + Proportion High-Use | 5 | -16.44 | 45.32 | 3.62 | 0.01 |
|  | 22 | Reverse Migratory Movements + Stopovers | 4 | -17.90 | 45.38 | 3.68 | 0.01 |
|  | 23 | Stopovers + Migration Days | 4 | -17.96 | 45.50 | 3.80 | 0.01 |
|  | 24 | Pre-nesting Duration + Migration Distance | 4 | -18.04 | 45.66 | 3.96 | 0.01 |
|  | 25 | Pre-nesting Duration + Migration Days | 4 | -18.06 | 45.69 | 3.99 | 0.01 |
|  | 26 | Reverse Migratory Movements + Stopovers + Proportion High-Use | 5 | -16.63 | 45.69 | 3.99 | 0.01 |
|  | 27 | Pre-nesting Duration + Reverse Migratory Movements | 4 | -18.06 | 45.69 | 3.99 | 0.01 |
|  | 28 | Pre-nesting Duration + Reverse Migratory Movements + Proportion High-Use | 5 | -16.65 | 45.74 | 4.04 | 0.01 |
|  | 29 | Migration Distance + Nest Initiation | 4 | -18.13 | 45.83 | 4.13 | 0.01 |
|  | 30 | Pre-nesting Duration + Stopovers + Proportion High-Use | 5 | -16.73 | 45.91 | 4.21 | 0.01 |
|  | 31 | Migration Distance + Stopovers | 4 | -18.17 | 45.91 | 4.21 | 0.01 |
|  | 32 | Pre-nesting Duration + Nest Initiation + Stopovers | 5 | -16.82 | 46.08 | 4.38 | 0.01 |
|  | 33 | Pre-nesting Duration + Migration Distance + Stopovers | 5 | -16.83 | 46.09 | 4.39 | 0.01 |
|  | 34 | Nest Initiation + Stopovers + Proportion High-Use | 5 | -16.83 | 46.11 | 4.41 | 0.01 |
|  | 35 | Pre-nesting Duration + Proportion High-Use + Migration Days | 5 | -16.85 | 46.13 | 4.43 | 0.01 |
|  | 36 | Migration Distance + Nest Initiation + Proportion High-Use | 5 | -16.87 | 46.17 | 4.47 | 0.01 |
|  | 37 | Pre-nesting Duration + Migration Distance + Nest Initiation + Proportion High-Use | 6 | -15.39 | 46.32 | 4.62 | 0.01 |
|  | 38 | Migration Distance + Reverse Migratory Movements + Proportion High-Use | 5 | -16.95 | 46.33 | 4.63 | 0.01 |
|  | 39 | Migration Distance + Proportion High-Use + Total Migration Days | 5 | -16.95 | 46.34 | 4.64 | 0.01 |
|  | 40 | Nest Initiation + Reverse Migratory Movements | 4 | -18.39 | 46.35 | 4.65 | 0.01 |
|  | 41 | Reverse Migratory Movements + Proportion High-Use + Migration Days | 5 | -17.03 | 46.50 | 4.80 | 0.01 |
|  | 42 | Migration Distance + Stopovers + Proportion High-Use | 5 | -17.07 | 46.58 | 4.88 | 0.01 |
|  | 43 | Migration Distance + Migration Days | 4 | -18.56 | 46.70 | 5.00 | 0.01 |
|  | 44 | Reverse Migratory Movements + Migration Days | 4 | -18.57 | 46.72 | 5.02 | 0.01 |
|  | 45 | Pre-nesting Duration + Migration Distance + Reverse Migratory Movements + Stopovers + Proportion High-Use | 7 | -13.91 | 46.72 | 5.02 | 0.01 |
|  | 46 | Migration Distance + Reverse Migratory Movements | 4 | -18.60 | 46.78 | 5.08 | 0.01 |
|  | 47 | Stopovers + Proportion High-Use + Migration Days | 5 | -17.21 | 46.86 | 5.16 | 0.01 |
|  | 48 | Pre-nesting Duration + Reverse Migratory Movements + Stopovers | 5 | -17.23 | 46.91 | 5.21 | 0.01 |
|  | 49 | Pre-nesting Duration + Nest Initiation + Reverse Migratory Movements + Proportion High-Use | 6 | -15.73 | 47.00 | 5.30 | 0.01 |
|  | 50 | Pre-nesting Duration + Migration Distance + Reverse Migratory Movements + Proportion High-Use | 6 | -15.81 | 47.16 | 5.46 | 0.01 |
|  | 51 | Pre-nesting Duration + Migration Distance + Stopovers + Proportion High-Use + Migration Days | 7 | -14.17 | 47.24 | 5.54 | 0.01 |
|  | 52 | Pre-nesting Duration + Migration Distance + Proportion High-Use + Migration Days | 6 | -15.87 | 47.28 | 5.58 | 0.01 |
|  | 53 | Pre-nesting Duration + Stopovers + Migration Days | 5 | -17.44 | 47.33 | 5.63 | 0.01 |
|  | 54 | Pre-nesting Duration + Nest Initiation + Reverse Migratory Movements | 5 | -17.50 | 47.45 | 5.75 | < 0.01 |
|  | 55 | Pre-nesting Duration + Migration Duration + Nest Initiation | 5 | -17.51 | 47.45 | 5.75 | < 0.01 |
|  | 56 | Pre-nesting Duration + Nest Initiation + Stopovers + Proportion High-Use | 6 | -15.96 | 47.46 | 5.76 | < 0.01 |
|  | 57 | Nest Initiation + Reverse Migratory Movements + Stopovers | 5 | -17.59 | 47.62 | 5.92 | < 0.01 |
|  | 58 | Nest Initiation + Reverse Migratory Movements + Stopovers + Proportion High-Use | 6 | -16.05 | 47.64 | 5.94 | < 0.01 |
| East | Breeding Attempt or Deferral |  |  |  |  |  |  |
|  | 1 | Migration Days | 3 | -18.27 | 43.49 | 0.00 | 0.17 |
|  | 2 | NULL | 2 | -19.67 | 43.80 | 0.31 | 0.14 |
|  | 3 | Arrival Date | 3 | -19.26 | 45.49 | 2.00 | 0.06 |
|  | 4 | Proportion High-Use + Migration Days | 4 | -18.07 | 45.81 | 2.32 | 0.05 |
|  | 5 | Stopovers | 3 | -19.54 | 46.04 | 2.55 | 0.05 |
|  | 6 | Arrival Date + Migration Days | 4 | -18.19 | 46.04 | 2.55 | 0.05 |
|  | 7 | Migration Distance + Migration Days | 4 | -18.21 | 46.09 | 2.60 | 0.05 |
|  | 8 | Reverse Migratory Movements + Migration Days | 4 | -18.24 | 46.14 | 2.65 | 0.04 |
|  | 9 | Stopovers + Migration Days | 4 | -18.27 | 46.20 | 2.71 | 0.04 |
|  | 10 | Stopovers | 3 | -19.66 | 46.28 | 2.79 | 0.04 |
|  | 11 | Proportion High-Use | 3 | -19.67 | 46.29 | 2.80 | 0.04 |
|  | 12 | Migration Distance | 3 | -19.67 | 46.30 | 2.81 | 0.04 |
|  | 13 | Arrival Date + Proportion High-Use | 4 | -19.16 | 47.99 | 4.50 | 0.02 |
|  | 14 | Arrival Date + Stopovers | 4 | -19.17 | 48.01 | 4.52 | 0.02 |
|  | 15 | Migration Distance + Arrival Date | 4 | -19.25 | 48.17 | 4.68 | 0.02 |
|  | 16 | Arrival Date + Reverse Migratory Movements | 4 | -19.25 | 48.18 | 4.69 | 0.02 |
|  | 17 | Migration Distance + Arrival Date + Proportion High-Use | 5 | -17.90 | 48.40 | 4.91 | 0.01 |
|  | 18 | Migration Distance + Stopovers | 4 | -19.50 | 48.67 | 5.18 | 0.01 |
|  | 19 | Arrival Date + Proportion High-Use + Migration Days | 5 | -18.05 | 48.71 | 5.22 | 0.01 |
|  | 20 | Stopovers + Proportion High-Use | 4 | -19.54 | 48.74 | 5.25 | 0.01 |
|  | 21 | Reverse Migratory Movements + Proportion High-Use + Migration Days | 5 | -18.07 | 48.74 | 5.25 | 0.01 |
|  | 22 | Stopovers + Proportion High-Use + Migration Days | 5 | -18.07 | 48.75 | 5.26 | 0.01 |
|  | 23 | Migration Distance + Proportion High-Use + Total Migration Days | 5 | -18.12 | 48.85 | 5.36 | 0.01 |
|  | 24 | Migration Distance + Arrival Date + Migration Days | 5 | -18.14 | 48.88 | 5.39 | 0.01 |
|  | 25 | Arrival Date + Reverse Migratory Movements + Migration Days | 5 | -18.14 | 48.90 | 5.41 | 0.01 |
|  | 26 | Migration Distance + Stopovers + Migration Days | 5 | -18.15 | 48.91 | 5.42 | 0.01 |
|  | 27 | Arrival Date + Stopovers + Migration Days | 5 | -18.17 | 48.96 | 5.47 | 0.01 |
|  | 28 | Reverse Migratory Movements + Proportion High-Use | 4 | -19.65 | 48.97 | 5.48 | 0.01 |
|  | 29 | Migration Distance + Reverse Migratory Movements | 4 | -19.65 | 48.98 | 5.49 | 0.01 |
|  | 30 | Migration Distance + Proportion High-Use | 4 | -19.67 | 49.00 | 5.51 | 0.01 |
|  | Breeding Success or Failure |  |  |  |  |  |  |
|  | 1 | Migration Days | 2 | -6.18 | 17.22 | 0.00 | 0.30 |
|  | 2 | NULL | 1 | -7.92 | 18.11 | 0.89 | 0.19 |
|  | 3 | Stopovers | 2 | -7.32 | 19.49 | 2.27 | 0.09 |
|  | 4 | Proportion High-Use | 2 | -7.50 | 19.87 | 2.65 | 0.08 |
|  | 5 | Stopovers + Migration Days | 3 | -6.12 | 20.08 | 2.86 | 0.07 |
|  | 6 | Proportion High-Use + Migration Days | 3 | -6.14 | 20.14 | 2.92 | 0.07 |
|  | 7 | Migration Distance + Migration Days | 3 | -6.17 | 20.18 | 2.96 | 0.07 |
|  | 8 | Migration Distance | 2 | -7.85 | 20.56 | 3.34 | 0.06 |
|  | 9 | Migration Distance + Stopovers | 3 | -6.89 | 21.63 | 4.41 | 0.03 |
|  | 10 | Stopovers + Migration Days | 3 | -7.23 | 22.30 | 5.08 | 0.02 |
|  | 11 | Migration Distance + Proportion High-Use | 3 | -7.24 | 22.34 | 5.12 | 0.02 |
| West | Breeding Attempt or Deferral |  |  |  |  |  |  |
|  | 1 | Arrival Date | 3 | -13.69 | 34.43 | 0.00 | 0.31 |
|  | 2 | Migration Distance + Arrival Date | 4 | -13.32 | 34.46 | 0.03 | 0.11 |
|  | 3 | Arrival Date + Migration Days | 4 | -13.44 | 36.71 | 2.28 | 0.1 |
|  | 4 | Arrival Date + Proportion High-Use | 4 | -13.48 | 36.78 | 2.35 | 0.1 |
|  | 5 | Arrival Date + Stopovers | 4 | -13.56 | 36.93 | 2.50 | 0.09 |
|  | 6 | Arrival Date + Reverse Migratory Movements | 4 | -13.69 | 37.20 | 2.77 | 0.08 |
|  | 7 | Migration Distance + Arrival Date + Migration Days | 5 | -13.16 | 39.18 | 4.75 | 0.03 |
|  | 8 | Migration Distance + Arrival Date + Proportion High-Use | 5 | -13.23 | 39.31 | 4.88 | 0.03 |
|  | 9 | Migration Distance + Arrival Date + Reverse Migratory Movements | 5 | -13.26 | 39.38 | 4.95 | 0.03 |
|  | 10 | Migration Distance + Arrival Date + Stopovers | 5 | -13.31 | 39.48 | 5.05 | 0.02 |
|  | 11 | Arrival Date + Stopovers + Proportion High-Use | 5 | -13.36 | 39.57 | 5.14 | 0.02 |
|  | 12 | Arrival Date + Proportion High-Use + Migration Days | 5 | -13.36 | 39.59 | 5.16 | 0.02 |
|  | 13 | Arrival Date + Stopovers + Migration Days | 5 | -13.38 | 39.61 | 5.18 | 0.02 |
|  | 14 | Arrival Date + Reverse Migratory Movements + Migration Days | 5 | -13.42 | 39.69 | 5.26 | 0.02 |
|  | 15 | Arrival Date + Reverse Migratory Movements + Proportion High-Use | 5 | -13.48 | 39.81 | 5.38 | 0.02 |
|  | Breeding Success or Failure |  |  |  |  |  |  |
|  | 1 | NULL | 1 | -9.56 | 21.50 | 0.00 | 0.34 |
|  | 2 | Proportion High-Use | 2 | -9.28 | 23.70 | 2.20 | 0.11 |
|  | 3 | Migration Distance | 2 | -9.35 | 23.85 | 2.35 | 0.11 |
|  | 4 | Stopovers | 2 | -9.38 | 23.90 | 2.40 | 0.10 |
|  | 5 | Migration Days | 2 | -9.44 | 24.02 | 2.52 | 0.10 |
|  | 6 | Migration Distance + Stopovers | 3 | -7.99 | 24.42 | 2.92 | 0.08 |
|  | 7 | Migration Distance + Proportion High-Use | 3 | -8.49 | 25.42 | 3.92 | 0.05 |
|  | 8 | Proportion High-Use + Migration Days | 3 | -9.06 | 26.56 | 5.06 | 0.03 |
|  | 9 | Stopovers + Migration Days | 3 | -9.17 | 26.79 | 5.29 | 0.02 |
|  | 10 | Stopovers + Proportion High-Use | 3 | -9.25 | 26.94 | 5.44 | 0.02 |
|  | 11 | Migration Distance + Migration Days | 3 | -9.27 | 26.99 | 5.49 | 0.02 |
|  | 12 | Migration Distance + Stopovers + Proportion High-Use | 4 | -7.29 | 27.07 | 5.57 | 0.02 |
|  |  |  |  |  |  |  |  |
